# Predictive prosthetic socket design: part 2—generating person-specific candidate designs using multi-objective genetic algorithms

**DOI:** 10.1007/s10237-019-01258-7

**Published:** 2019-11-18

**Authors:** J. W. Steer, P. A. Grudniewski, M. Browne, P. R. Worsley, A. J. Sobey, A. S. Dickinson

**Affiliations:** 1 Faculty of Engineering and Physical Sciences, University of Southampton, Southampton, UK; 2 Faculty of Environmental and Life Sciences, University of Southampton, Southampton, UK

**Keywords:** FEA, Amputation, Residual limb, Optimisation

## Abstract

In post-amputation rehabilitation, a common goal is to return to ambulation using a prosthetic limb, suspended by a customised socket. Prosthetic socket design aims to optimise load transfer between the residual limb and mechanical limb, by customisation to the user. This is a time-consuming process, and with the increase in people requiring these prosthetics, it is vital that these personalised devices can be produced rapidly while maintaining excellent fit, to maximise function and comfort. Prosthetic sockets are designed by capturing the residual limb’s shape and applying a series of geometrical modifications, called rectifications. Expert knowledge is required to achieve a comfortable fit in this iterative process. A variety of rectifications can be made, grouped into established strategies [e.g. in transtibial sockets: patellar tendon bearing (PTB) and total surface bearing (TSB)], creating a complex design space. To date, adoption of advanced engineering solutions to support fitting has been limited. One method is numerical optimisation, which allows the designer a number of likely candidate solutions to start the design process. Numerical optimisation is commonly used in many industries but not prevalent in the design of prosthetic sockets. This paper therefore presents candidate shape optimisation methods which might benefit the prosthetist and the limb user, by blending the state of the art from prosthetic mechanical design, surrogate modelling and evolutionary computation. The result of the analysis is a series of prosthetic socket designs that preferentially load and unload the pressure tolerant and intolerant regions of the residual limb. This spectrum is bounded by the general forms of the PTB and TSB designs, with a series of variations in between that represent a compromise between these accepted approaches. This results in a difference in pressure of up to 31 kPa over the fibula head and 14 kPa over the residuum tip. The presented methods would allow a trained prosthetist to rapidly assess these likely candidates and then to make final detailed modifications and fine-tuning. Importantly, insights gained about the design should be seen as a compliment, not a replacement, for the prosthetist’s skill and experience. We propose instead that this method might reduce the time spent on the early stages of socket design and allow prosthetists to focus on the most skilled and creative tasks of fine-tuning the design, in face-to-face consultation with their client.

## Requirement of automation in design of prosthetics

Approximately 40 million people globally require access to prosthetic or orthotic services (Eklund and Sexton 2017). Prosthesis–human interface design aims to maximise comfort and functionality for people with amputations, towards ambulatory rehabilitation. This is commonly provided through a prosthetic socket, which is designed through geometric modifications to the captured shape of the residual limb, known as rectifications, to create a desired pattern of load transfer. This is currently an iterative process performed by a highly skilled prosthetist, who manages the residuum’s changing size, shape, soft tissue healing and biomechanical adaptation. Indeed, due to these factors, the development of a definitive socket takes a considerable period of time. Prosthetic limb users require lifelong access to prosthetics services, and in the UK the annual cost of prosthesis provision and care is over £2800 per patient (Kerr et al. 2014). This includes the replacement of prosthetic limb components typically every 2 to 5 years. Skilled prosthetists take many years to train to a high standard, and often prosthetic users develop relationships with their preferred clinician to maintain socket comfort. However, there are limited numbers of these highly skilled individuals and practice efficiencies are required in the face of growing clinical demand. Researchers have considered mechanisms for employing quantitative prediction in the socket design process (Goh et al. 2005; Colombo et al. 2013), although at present these work to a single design target for a single individual and have not entered conventional clinical use.

In Part 1 of this study (Steer et al. 2019), a kriging-based surrogate model was generated for a parametric FE model of a population-based transtibial residual limb and accompanying total surface bearing (TSB) socket design. This enabled the prediction of biomechanical relationships between the residual limb morphology and prosthetic socket design, while reducing the computational cost of each new prediction by six orders of magnitudes (1.6 ms vs. 30 min). The simplified total surface bearing socket design was defined parametrically from the limb’s neutral shape, by reducing the cross-sectional area along its length with three points at the proximal, mid and distal regions of the socket. However, within a clinical setting, the socket design process is substantially more nuanced. There are several different design philosophies, all with different intended residual limb load transfer mechanisms. The classic patella tendon bearing (PTB) socket design was developed in 1957 and is still commonly used in-clinic today (Radcliffe 1962). This socket design aims to apply pressure over load-tolerant areas of the limb such as the patella tendon, and off-load pressure-sensitive regions such as the anterior tibia, fibula head and residuum tip. Other sockets include the Kondylen-Bettung Münster (KBM) which provides supracondylar suspension in addition to features consistent with the patella tendon bearing design (Kuhn 1966), and hydrostatic sockets (Murdoch 1964) such as the PCAST system (Lee et al. 2000; Goh et al. 2003; Goh et al. 2004; Laing et al. 2017; Laing et al. 2018) which uses a pressurised fluid as a medium to form the shape of the socket with the aim of achieving minimal residuum surface pressure gradients with less manual intervention. More recently, total surface bearing sockets, which were proposed in 1987, are used to generate near-total contact in between the residual limb and the socket (Staats and Lundt 1987). In theory, this should maximise the contact area between the residual limb and prosthetic socket and the uniformity of pressure across the surface of the residual limb, thereby minimising potentially harmful pressure gradients (Hachisuka et al. 1998).

Despite the fundamental differences in the load distribution between these socket designs, they can potentially all deliver satisfactory outcomes for prosthesis users. There is substantial research into quantifying the biomechanical differences between these socket designs, which is comprehensively reviewed by Safari and Meier in (2015). Their systematic review concluded that ‘the included studies only had low to moderate methodological rigour’, thus demonstrating the difficulties in defining biomechanical guidelines for the highly dynamic environment of the residual limb—prosthetic socket system, or selection of the preferred socket type for a particular individual or situation. One possible reason for the difficulty in establishing the definitive guidelines of these different socket types is that they are defined primarily by design intent, rather than quantitative rules. This effect has been illustrated for a simple total surface bearing socket using parametric FEA (Steer et al. 2019), and it is almost certain the within-type variability would be increased for more complex designs. We propose that there is a large potential to enhance the evidence base behind this clinical challenge, allowing prosthetists to develop, critique and share their own expertise and decision-making, making more effective use of their valuable design and consultation time. A key and relatively unexplored possibility is to apply automated search algorithms to explore designs prior to optimisation for the individual.

Optimisation algorithms are common in many areas of engineering. They are used as concept design methods, providing an initial product which engineers can use as a starting point and to increase the proportion of their time spent on creatively solving complex problems. In addition, they provide a visualisation for how these changes will affect the final product’s performance, allowing a greater understanding of the design space which can be put to use in the more detailed stages of the process. A choice of potential candidate designs can be provided to the decision maker, which weight the objectives differently, for example putting more load on one region of a structure and removing it from another, and therefore give a range of performances. This requires algorithms capable of multi-objective optimisation that provide a rapid convergence on the global optimum while retaining a high diversity of the search, to ensure that the entire search space is investigated and that the focus is not upon local optima. Many methodologies have been developed, and state-of-the-art research focuses on improvements in diversity or convergence.

This paper employs optimisation algorithms to generate personalised ‘candidate’ prosthetic socket designs for the first time. This is applied to the transtibial case, which is the most common major lower limb amputation and where most clinical success has been achieved with associated CAD/CAM socket design and fabrication tools. Different design problems require different optimisation processes. The aim is therefore to determine appropriate methods for the automated application of candidate socket rectifications, collating the state of the art in biomechanical analysis of prosthesis–limb interfaces, surrogate modelling and optimisation. Genetic Algorithms are chosen due to their ability to effectively search large and complex design spaces, which is the problem presented by the continually variable distribution of possible limb–socket shape rectifications. These methods rely on thousands of function calls, and using FE models would not be feasible beyond single cases due to the time required for each simulation. However, by leveraging the speed increases of the surrogate model (Steer et al. 2019), it becomes technically feasible to perform automated socket optimisation based upon structural analysis of the limb–prosthesis system. This provides the motivation for the current study, to perform a first-of-kind, subject-specific, multi-objective design optimisation of the prosthetic socket using the previously reported surrogate model. The result will be a series of personalised ‘candidate’ transtibial prosthetic socket designs, to which the prosthetist would add local modifications based upon their knowledge and conventional patient consultation. Finally, equipped with these results, a prosthetist would then further refine these concepts to achieve a desired pattern of prosthesis–limb load transfer, by using these designs to augment their experience-based decision-making.

## Optimisation of transtibial prosthetic sockets

### Population-based surrogate model

**Fig. 1:**
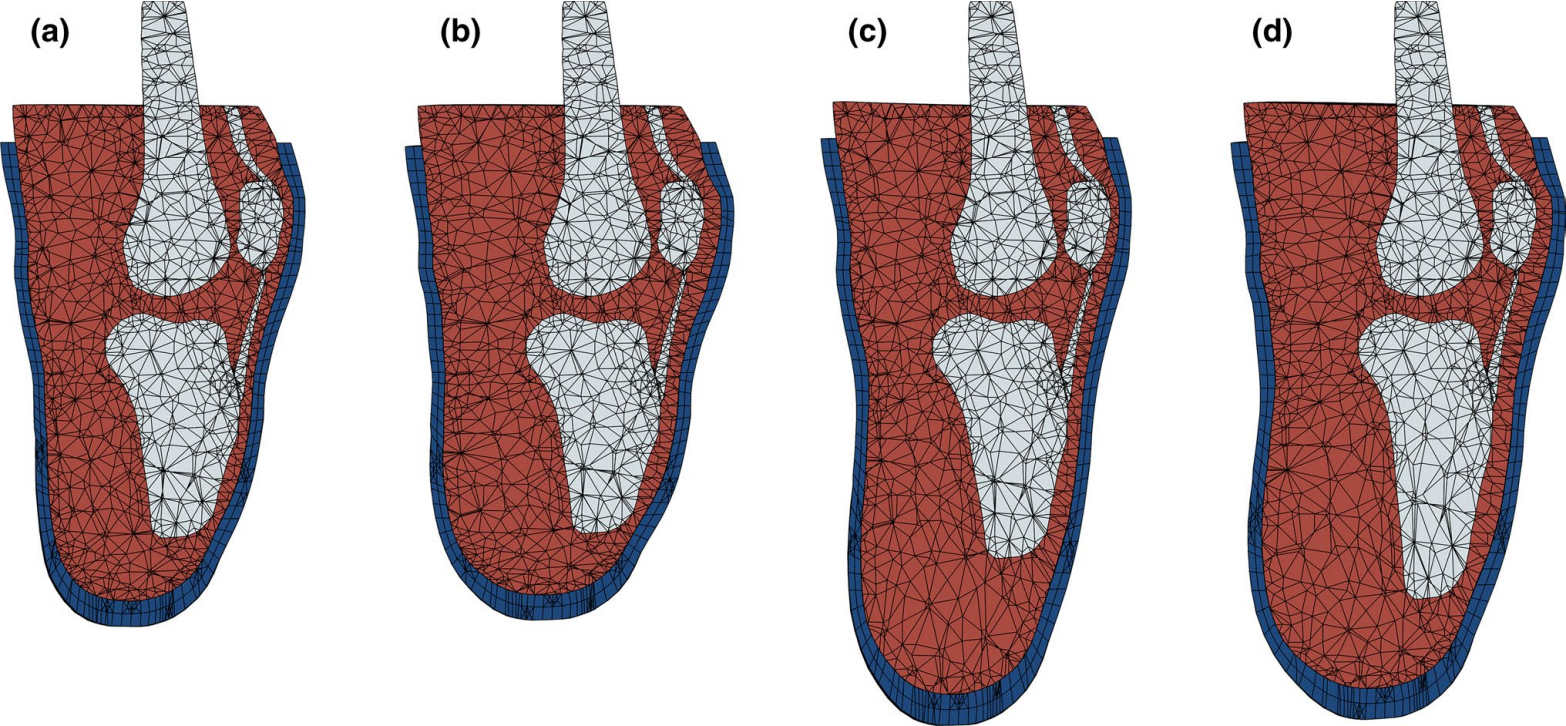
Sagittal sections through equivalent residuum FE models for the four virtual people. Blue indicates the liner, red the soft tissue, and grey the bones. The prosthetic socket is not shown

A detailed description of the population-based surrogate model is found in Part 1 of this paper (Steer et al. 2019). In short, a generic residual limb was generated by producing a volume mesh from an MRI scan and imposing radial basis function mesh morphing to apply parametric variation in residuum length and profile (conical to bulbous) obtained from principal modes of variation from a population of 3D surface scans. These were varied by ± 1 $\sigma$ (standard deviation) about the mean length and profile in the statistical shape model (SSM). Furthermore, internal parametric variation of the relative tibia length (i.e. distal soft tissue coverage) from − 15% to + 30% of the tibia length from the MRI scan, and soft tissue material properties between stiff, flaccid muscle and contracted muscle (Palevski et al. 2006; Portnoy et al. 2009; Hoyt et al. 2008) were applied. The soft tissue was assigned a neo-Hookean material to capture the nonlinear behaviour of the soft tissue. The present surrogate model implementation investigates the effects of socket design variation for four synthetic ‘virtual’ people sequentially by selecting exemplar values for the model’s residuum variability parameters (Table 1, Fig. 1). These cases were chosen as being close to the models’ population extremes while remaining within the bounding box of the sampling plan, to avoid extrapolating beyond the surrogate. These meshes were imported into the finite element solver (ABAQUS 6.14, Dassault Systèmes, Vèlizy-Villacoublay, France). The socket was donned under displacement control and loaded uniaxially to 400 N to simulate a two-leg stance. The resultant pressure and soft tissue strain outputs from 75 simulations were used to construct a kriging surrogate model for each virtual person, enabling a function call to be made in ~ 2 ms.

**Table 1:** Parameters of the four cases extracted from the parametric residual limb model

Virtual person	Residuum length, $v_{1}$	Residuum profile, $v_{2}$	Tibia length, $v_{3}$	Soft tissue initial modulus, $v_{4}$
A	$-\, 0.8 \sigma$ (Short)	$-\,0.8 \sigma$ (Bulbous)	$+\, 20\%$ (Long)	$40\;{\text{kPa}}$ (Soft)
B	$-\,0.8 \sigma$ (Short)	$+\,0.8 \sigma$ (Conical)	$-\,5\%$ (Short)	$50 \;{\text{kPa}}$ (Stiff)
C	$+\, 0.8 \sigma$ (Long)	$-\,0.8 \sigma$ (Bulbous)	$+\,20\%$ (Long)	$40\;{\text{kPa}}$ (Soft)
D	$\mp 0.8 \sigma$ (Long)	$+\,0.8 \sigma$ (Conical)	$-\,5\%$ (Short)	$50 \;{\text{kPa}}$ (Stiff)

Soft tissue initial modulus corresponds to the initial stiffness of the applied neo-Hookean hyperelastic material model

**Table 2:** Parameters and limits of the parametric socket design

Socket rectification variable name	Lower bound	Upper bound
Proximal press fit	− 2%	+ 6%
Mid press fit	− 2%	+ 6%
Distal press fit	− 2%	+ 6%
Patella tendon bar	0 mm	6 mm
Fibula head relief	0 mm	6 mm
Tibial crest	0 mm	6 mm

### Parametric socket design

**Fig. 2:**
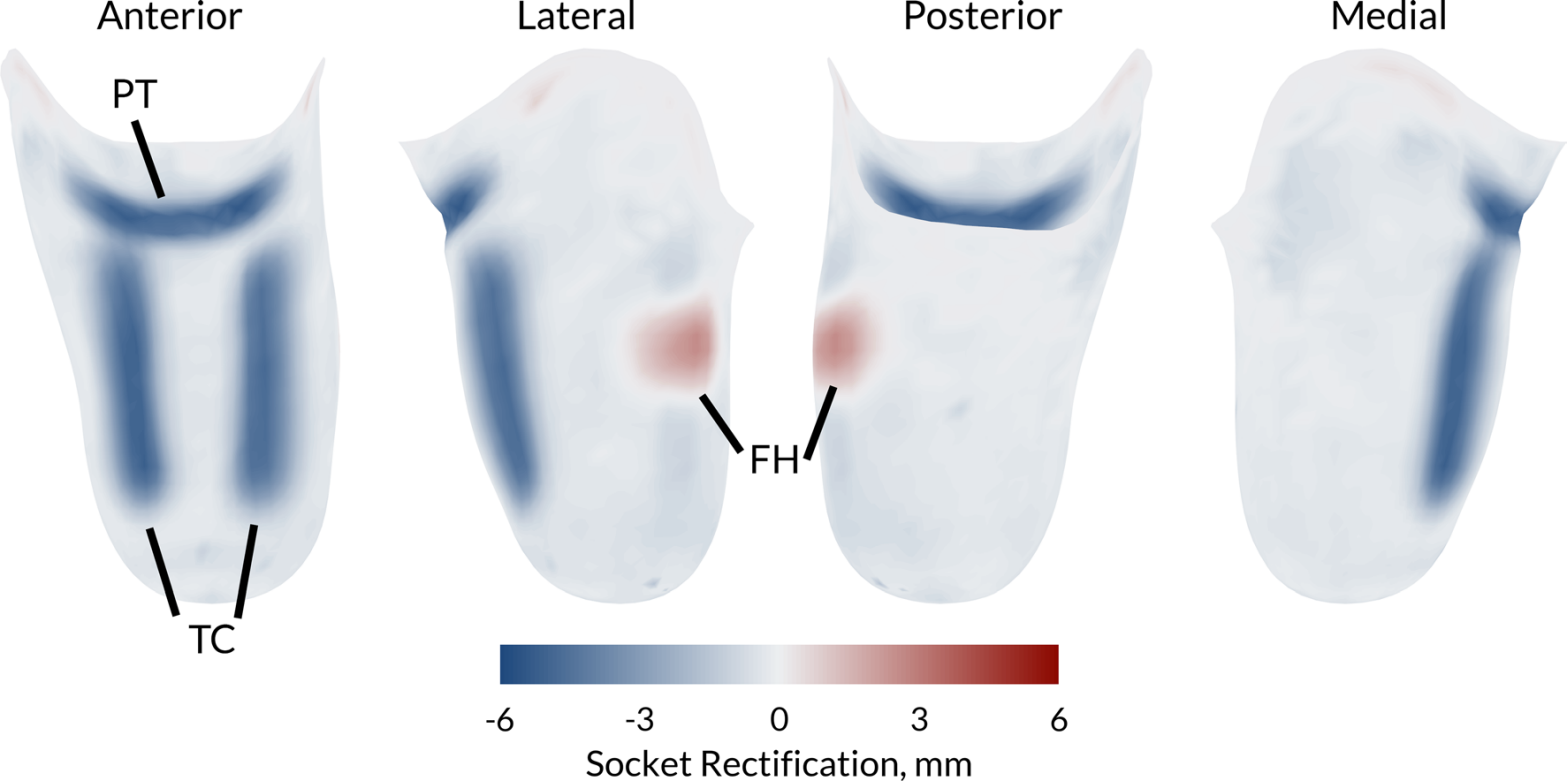
Rectification maps of the patella tendon bearing socket design at the maximum values of patella tendon bar (PTB), fibula head (FH) relief and tibial crest (TC) rectifications. The figure demonstrates the resulting socket shape change once the control nodes have been displaced and explains the convention directions of each rectification type (FH vs. PT and TC)

In the preceding work (Steer et al. 2019), a simplified, three-parameter total surface bearing socket design was used. This model enabled control of the socket press fit by reducing its cross-sectional area through a B-spline function with proximal, mid and distal control points. The three variables were constrained between − 1% and 3% by cross-sectional area reduction. The present study’s socket design was extended to include the localised rectifications observed in patella tendon bearing sockets. Control points were generated over the fibula head, patella tendon and either side of the tibial crest (Fig. 2). These localised rectifications were applied using the same radial basis function mesh morphing algorithm detailed above, by radially displacing the control points between 0 and 6 mm.

### Optimisation via genetic algorithms

Genetic algorithms (GA) are population-based multi-objective solvers inspired by the principles of Darwinian evolution (Goldberg and Holland 1988). In a simple Genetic Algorithm, a set of potential solutions, called individuals, reproduce via an evolutionary-like process. Each individual contains set of decision variables, called chromosomes, with an initial population with variables that are usually assigned randomly. The fitness of each individual can be evaluated according to some predefined objectives. After this step individuals are then chosen for reproduction, and according to the principles of natural selection, the fitter individuals have significantly higher chances of reproducing than those with a low fitness. Offspring are generated from the selected parents using crossover and mutation processes. During crossover, the chromosomes of the offspring are produced by mixing the genes of the parents, providing convergence and diversity. In the mutation step, the offspring’s genes have a small chance to be randomly modified, improving the population diversity. Finally, the old population becomes extinct and is replaced by the new generation, with the new generation being fitter, on average, than the parent generation. This process continues until the predefined termination condition is met, often specified as a maximum number of objective functions calls or total calculation time.

Many competing genetic algorithms have been developed, each introducing novel mechanisms to increase the convergence rates and diversity of the search. In the current state of the art of Gas, there is particular emphasis on specialist solvers. According to the ‘no free lunch’ theorem (Wolpert and Macready 1997), a specialist solver exhibits high performance on a narrow set of problems but its performance will rapidly decline when outside of this set. Therefore, a suitable methodology has to be selected with respect to the particular problem’s characteristics in order to avoid poor performance. The optimisation problem characteristics and their difficulty are defined by the topology of the search and objective spaces, number of local optima and the applied constraints. If the problem characteristics are not known, then more than one GA methodology should be applied as their performance can differ drastically. This will provide more reliable results and allow an evaluation of the problem’s difficulty and its dominant characteristics (Wang et al. 2018). In the case presented in this paper, no knowledge about the characteristic of the problem is available a priori, except that no constraints are used. However, this is not sufficient to choose a single properly adjusted optimiser. Therefore, five different Genetic Algorithms are compared: NSGA-II as the most commonly utilised Genetic Algorithm which retains a high diversity of search and has had much success in the applied literature (Deb et al. 2002); MOEA/D as the most proficient algorithm for unconstrained problems (Zhang and Li 2007); MTS as an aggregation of a Genetic Algorithm and a local-search method which provides improved convergence (Tseng and Chen 2007); cMLSGA and HEIA as the general-type GAs that exhibit high performance across wide range of problem types and therefore higher robustness (Lin et al. 2016; Grudniewski and Sobey 2019). HEIA is more dominant in scenarios where convergence is more important and cMLSGA provides a higher diversity of search. The detailed principles of working and parameter settings of each methodology can be found in their respective publications.^1^ All the tests were performed over 30 separate runs, with 50,000 fitness function evaluations as a termination criterion. Multiple runs must be performed in order to assure the robustness of the method and the best likelihood of identifying the true Pareto Front. Different population sizes were tested and 600 individuals were utilised as the best for NSGA-II, MTS, MOEA/D and HEIA, while cMLSGA utilised 1800 as it requires significantly higher population sizes (Grudniewski and Sobey 2018).

The socket design process presented in our prior work (Steer et al. 2019) can be framed as a formal engineering design optimisation problem. In this case the individual socket rectifications function as design parameters across a multi-dimensional input space, and the resultant pressure and soft tissue strain fields are formulated as the objective functions. The locations across the limb for the objective functions were selected because they are known to be load-intolerant (Radcliffe 1962). It was predicted that the introduction of a peripheral press fit around the main body of the residuum will allow load transfer through the longitudinal shear forces and thus reduce the residuum tip pressure, at the expense of pressure concentrations over the bony prominences of the tibial tuberosity and fibula head. Four state variables were defined: the pressure over the residuum tip ($f_{1}$), the tibial tuberosity ($f_{2}$), the fibula head ($f_{3}$) and the soft tissue strain around the distal tibia ($f_{4}$). These model outputs can be described as competing fitness functions, indicating proximal and distal loading, defined as ${\text{FF}}1 = f_{1} + f_{4}$ and ${\text{FF}}2 = f_{2} + f_{3}$. These were evaluated using the surrogate model developed previously (Steer et al. 2019) for the four synthetic people defined in Table 1.

One of the issues with multi-objective optimisation is the comparison of the results obtained by different methods. The visual comparison is limited, only providing useful information when the performance of two solvers differs drastically. Otherwise, the points will overlap making objective comparison near impossible. Therefore, multiple quality indicators have been developed (Li and Yao 2018). Most of them are able to indicate the performance in both convergence and diversity of the solutions. However, each of them has certain drawbacks or biases and it is common practice to utilise more than one indicator (Li and Yao 2018). In this paper the inverted generational distance (IGD) and hypervolume (HV) were chosen as indicators. IGD measures the average Euclidean distance between each point in a real Pareto Optimal Front (POF) and the closest solution in the obtained set. Lower values indicate better convergence and uniformity of the points and are calculated according to Eq. 1:

1 $${\text{IGD}}\left( {A,P^{*} } \right) = \frac{{\mathop \sum \nolimits_{{\nu \in P^{*} }} {\text{d}}\left( {\nu ,A} \right)}}{{\left| {P^{*} } \right|}},$$

where *P*^∗^ is a set of uniformly distributed points along the true PF, A is the approximate set to the POF, which is being evaluated and d(*ν*, *A*) is the minimum Euclidean distance between the point *ν* and points in *A*.

However, this IGD shows poor performance in determining the diversity of a population when the Pareto Front population is small. HV is calculated as the volume of an objective space between a predefined reference point and the obtained solutions where higher values are preferred (Li and Yao 2018). This indicator has a stronger focus on the diversity and boundary points. Most indicators require a predefined reference Pareto Optimal Front that illustrates the ideal set of solutions. However, in cases where the optimal answer is not known the utilisation of these indicators can be problematic. A solution is to calculate a reference Pareto Optimal Front using the non-dominated selection of Pareto Optimal Fronts achieved by every algorithm when performing multiple runs, or performing a few test runs with significantly higher numbers of iterations than that utilised for comparison (Wang et al. 2018). In this paper both are applied, and a combined non-dominated front obtained by brute force from all 6 Genetic Algorithms after 300,000 fitness function evaluations was used to determine the success of the algorithm.

## Results

**Fig. 3:**
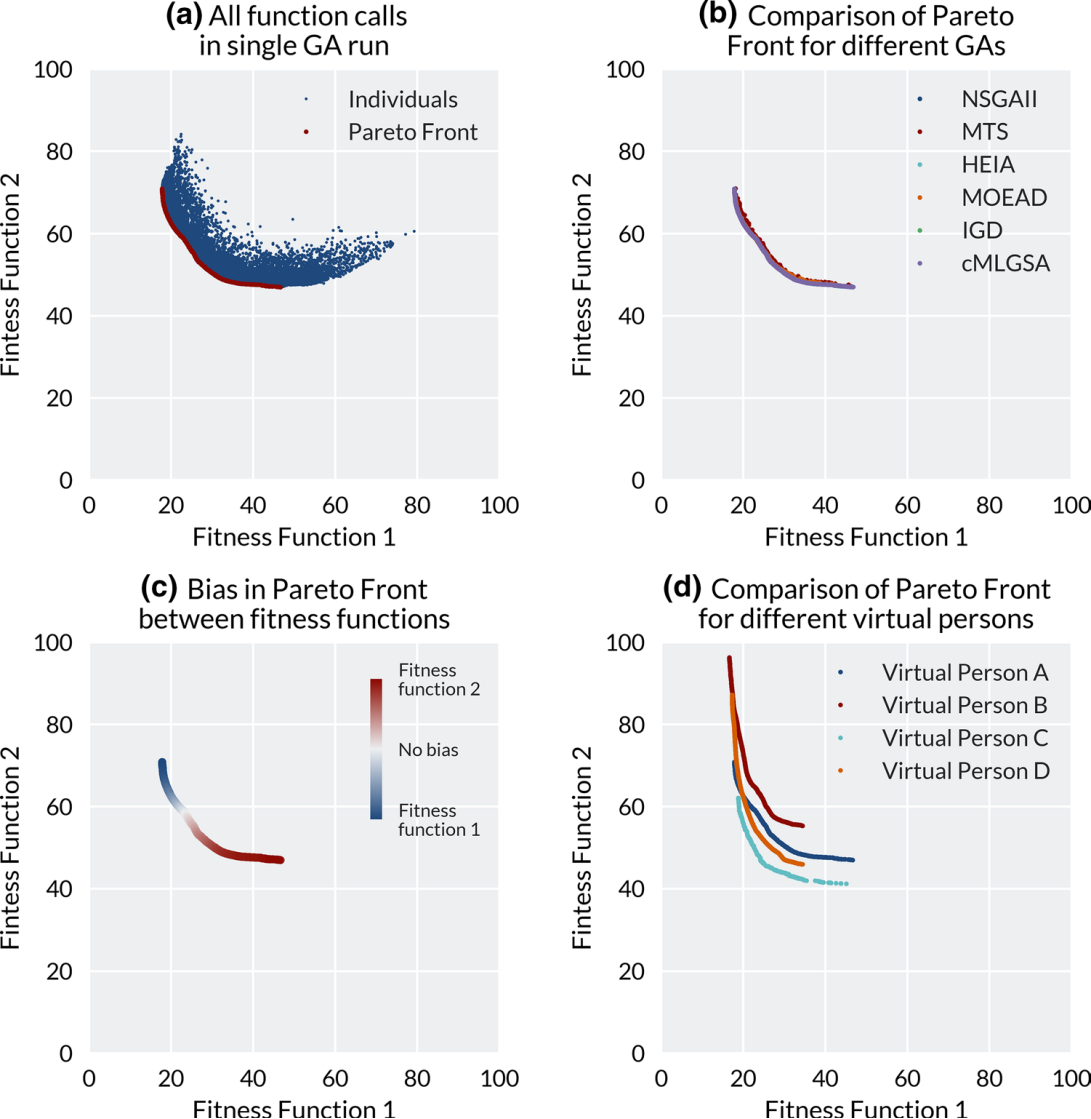
Analysis of the Pareto fronts from the multi-objective optimisation. **a** Individuals from a single run of the HEIA optimisation for Person A, with all individuals plotted in blue and Pareto front in red. **b** Comparison of the generated PFs for the six different GAs tested on Person A. **c** Bias along the Pareto front between the two fitness functions, with ‘no bias’ defined as the minimum distance from the origin to the normalised Pareto Optimal Front, with blue indicating bias towards FF1 and red towards FF2. **d** Comparison of the Pareto Fronts for the four different People when using HEIA

A single Genetic Algorithm run with a maximum of 50,000 function calls was computed in approximately 30 min, where Fig. 3a shows the individuals evaluated over this lifetime and the final Pareto Front. Comparing the different genetic algorithms, it was observed that the shape of the Pareto Optimal Fronts remains consistent. Therefore, visual comparison only shows that all of the methodologies exhibit similar performance and it is not possible to unanimously choose the best methodology (Fig. 3b). The bias between Fitness Functions FF1 and FF2 along the normalised Pareto Optimal Front is visualised in Fig. 3c. The reason the no-bias point is not in the middle of the front is due to the longer ‘tail’ when minimisation is biased towards FF2 (minimising proximal bony prominence loading), compared with bias towards FF1 (minimising distal tip loading). It was also observed that while the minima of FF1 for all individuals plateaued at just below 20 (unitless), the minima of FF2 were different for all of the virtual people (Fig. 3d). The minima of the short, conical limb of person B and the long, bulbous limb of person C plateaued at FF2 = 55 kPa and 40 kPa, respectively.

**Fig. 4:**
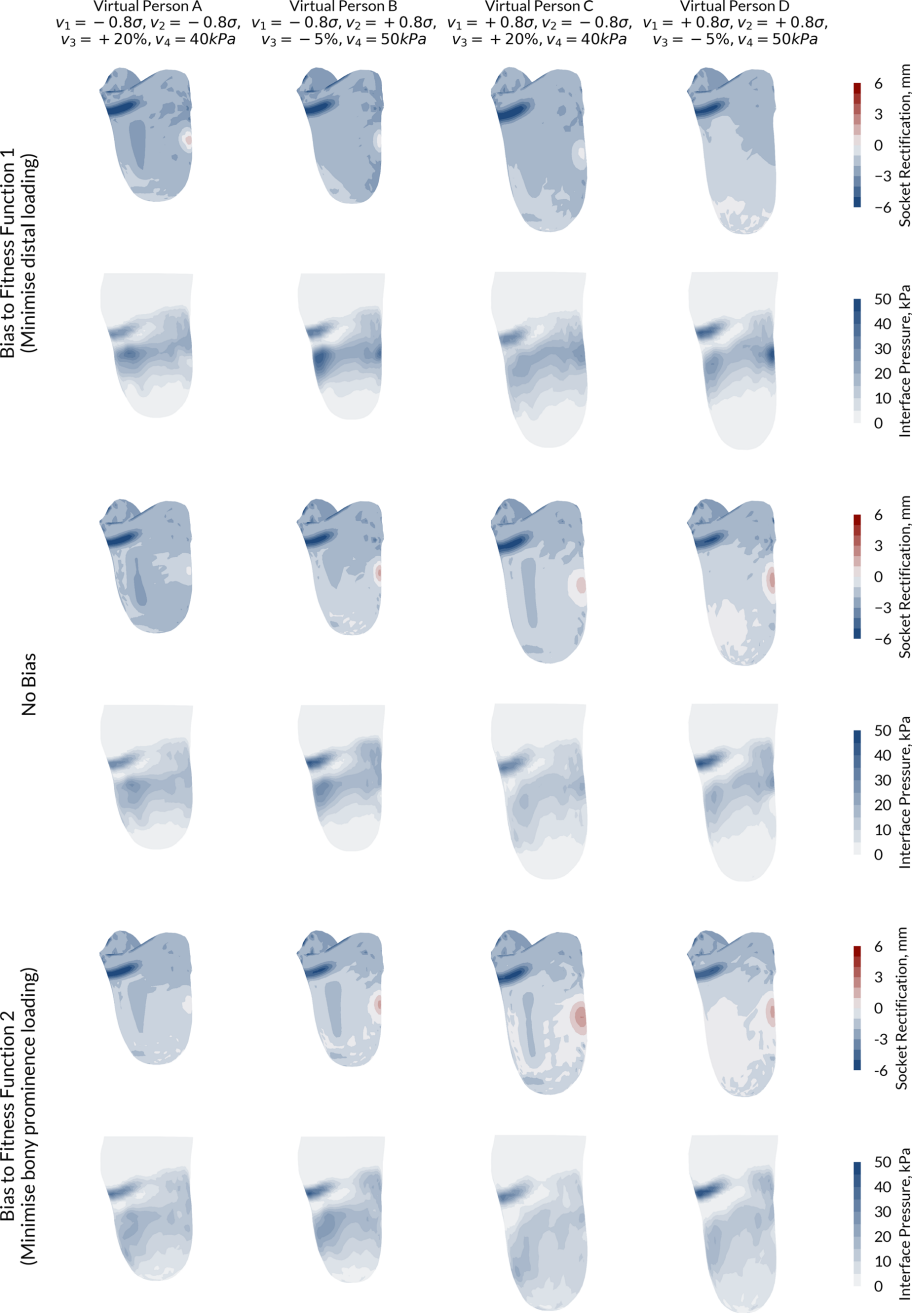
Optimal socket designs and corresponding predicted pressure maps for the four different virtual people at the two ends of the POF, i.e. biased towards minimising distal tip loading (top) and minimising proximal bony prominence loading (bottom), and the design with no bias (centre)

From visualisation of the sockets at either end of the Pareto Front, as well as the neutral case, consistencies in design emerged across the four people (Fig. 4). When the optimiser was biased towards FF1 (minimising tip loading), designs of higher press fit which off-loaded the residuum tip emerged from the Genetic Algorithm. For person B (Fig. 4b) and person D (Fig. 4d), pressure hotspots were generated where there was little soft tissue coverage over the proximal bony prominences. When the model was biased towards FF2, sockets with higher fibula head relief evolved in order to off-load over this region.

**Fig. 5:**
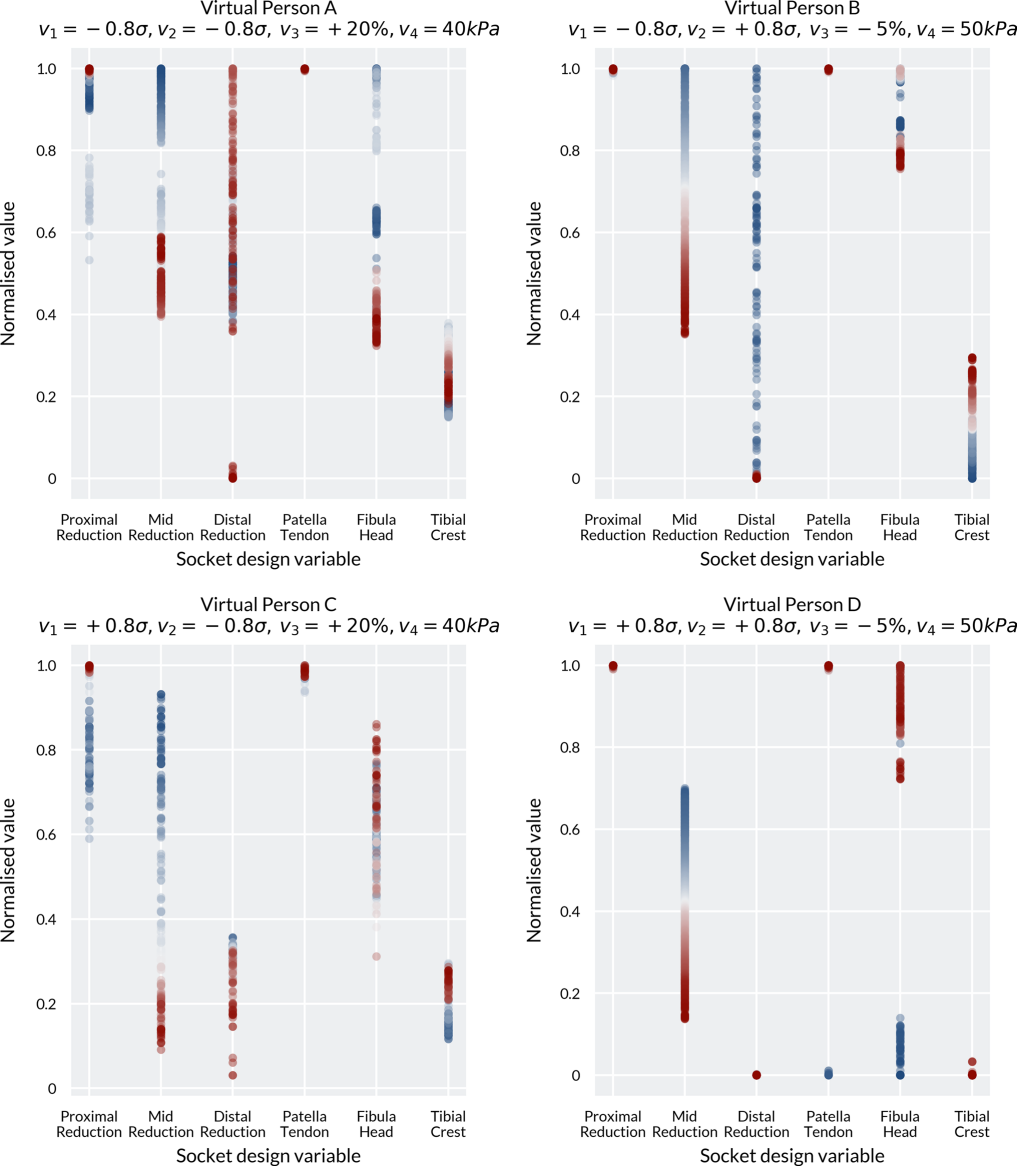
Comparison of how the socket design variables (see Table 2) changed between the four cases along the Pareto Optimal Front. Blue denotes a bias towards FF1 (distal loading), while red denotes bias towards FF2 (proximal loading)

Trends in the designs can be observed between the competing fitness functions by visualising how the optimal socket designs change along the Pareto Optimal Front (Fig. 5). Across all virtual people, the patella tendon bar variable converged at the constraint maximum of 6 mm for almost all of the points along the Pareto Optimal Front. The exception was in Person D, with the longest and thinnest residual limb. When the optimiser was biased towards FF1, a few designs evolved with the patella tendon bar rectification at the 0 mm lower limit. This was offset by removal of the fibula head relief to ensure that the pressure over the residuum tip is still minimised. A clear trend for all virtual people was in the mid reduction in the socket, where the press fit decreases along the Pareto Optimal Front from FF1 (with the aim of minimising the distal loading) to FF2 (with the aim of minimising the proximal loading). By reducing the press fit, the pressure over the bony prominences and the peripheral shear both decreased, resulting in an increase in distal tip pressure and soft tissue strain.

The performance of different methodologies was evaluated using the proposed indicators, IGD and HV and presented in the form of rankings with average values and standard deviations (Table 3). In this case the algorithms all performed in the same manner for IGD and HV. HEIA and cMLSGA were the best performing algorithms and MOEA/D and MTS performed the worst. However, the relatively similar performances of all five algorithms indicate that the complexity of the presented cases is low. The final Pareto Front was continuous and there were no constraints, which led to convergence-dominated HEIA having the best performance, over cMLSGA and NSGA-II. MOEA/D and MTS perform less well. However, this may be due to a lack of hyperparameter tuning to the particular problem. These two algorithms are dependent on a number of parameters which must be optimised for each problem, and in the present work the authors used default values described in the algorithms’ original papers. The MOEA/D and MTS algorithms may perform better once tuned, now that a priori knowledge has been developed, but the present results indicate the caution with which these algorithms should be used.

**Table 3:** Ranking of different genetic algorithms using HV and IGD as the performance indicators

Rank	1	2	3	4	5
IGD					
Algorithm	HEIA*	cMLSGA*	NSGA-II*	MOEA/D*	MTS
Average	0.029349	0.057565	0.1384	0.281601	0.590279
(S.D.)	0.001741	0.001553	0.139218	0.158822	0.049102
HV					
Algorithm	HEIA*	cMLSGA	NSGA-II*	MOEA/D	MTS
Average	0.174846	0.174475	0.174461	0.174094	0.168158
(S.D.)	0.000027	0.000039	0.000288	0.000214	0.000464

*indicates if the results are significantly different to the next lowest rank, using the Wilcoxon’s rank sum with a 0.05 confidence

**Fig. 6:**
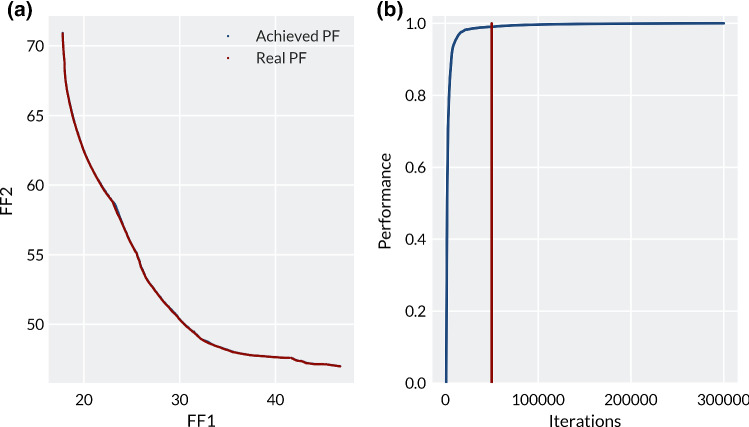
**a** The comparison of Pareto Fronts from Virtual Person 1, achieved using HEIA over 50,000 iterations (‘achieved’) and 300,000 iterations (‘real’). **b** The performance of HEIA over 300,000 iterations on Person 1. 0 is the starting population, and 1 is the best attainable set of solutions, based on the IGD values, and the red line indicates the number of function calls utilised in this study

In order to better understand the complexity of the problem and to check whether the best possible set of solutions has been found, a set of 5 runs with 300,000 total iterations was conducted on each virtual person, utilising HEIA. In this case hardly any difference was observed between 50,000 and 300,000 iterations. Figure 6a shows some very slightly higher uniformity and diversity of the points in the high FF1 bias region with 300,000 iterations. When comparing the performance over the number of generations in Fig. 6b, virtually no improvement in performance can be seen after 50,000 generations and the highest performance gain occurred before 25,000 iterations. The low possible performance increase beyond 50,000 iterations in this case would not justify conducting optimisation of this problem with higher values, unless the virtual person is suspected to benefit from an extreme reduction in pressure over the residuum tip and the soft tissue strain around the distal tibia (FF1 bias).

## Discussion

This study aimed to explore a range of potential concepts for transtibial socket design using FE modelling, surrogate modelling and GA-based optimisation techniques, to provide a quantitatively informed starting point for the prosthetist when designing a bespoke prosthetic socket.

Exploring the parametric socket design space demonstrates that biomechanical objective functions are in competition and illustrates the challenges associated with defining the ‘best’ socket design solution. As explored in our previous work (Steer et al. 2019), by increasing the socket press fit, particularly in the mid-section, an increase in longitudinal shear around the main body of the residual limb was predicted. This resulted in a pressure reduction at the residuum tip coupled to a reduction in the internal strain around the distal tibia. By oversizing the socket (i.e. negative press fit), these peripheral shear forces were not generated, thereby increasing the distal pressure and soft tissue strain. These represent the competing fitness functions inherent in prosthetic socket design.

Introduction of the patella tendon bar and tibial crest rectifications provided an alternative method of off-loading the residuum tip beyond a uniform press fit. Fibula head relief is predicted to be effective in reducing the high pressure that was observed over this bony prominence for the total surface bearing socket designs, thus reiterating the importance of localised shape change beyond applying gross scaling to the limb shape (Goh et al. 2003).

The sockets that emerged from the Genetic Algorithm exhibited features of both total surface bearing (TSB) and patella tendon bearing (PTB) manual socket design philosophies. One consistent feature along the Pareto Front for all virtual people was the patella tendon bar rectification variable, which saturated at its maximum limit. This is because no optimisation cost was associated with applying pressure over this region, which the Genetic Algorithm exploited to off-load the high-cost residuum tip region. This effect is observed clinically for the patella tendon bearing socket where prosthetists produce a marked rectification over the patella tendon to leverage its load bearing capacity. Although load tolerant, there clearly would be a load threshold for injury at the patella tendon, so with enhanced spatial data of load tolerance across these key residuum locations (Bramley et al. 2019) an additional constraint of maximal patellar tendon pressure could be included in the optimisation problem.

Along the Pareto Front of the best solutions, trends were predicted as the bias of the optimised socket varied between the two fitness functions. When fitness function 1 was dominant and the Genetic Algorithm aimed to minimise pressure and soft tissue strain at the residuum tip, sockets with high levels of mid-height press fit emerged from the model. Conversely, when fitness function 2 was dominant and the Genetic Algorithm aimed to minimise pressure over the proximal bony prominences, the global press fit was reduced and local relief over the fibula head was increased. The sockets which minimised residuum tip pressure (FF1-biased) exhibited characteristics associated with a total surface bearing socket design, while the patella tendon bearing rectifications were dominant when minimising pressure over bony prominences (FF2-biased). While it is difficult to validate these findings from the current literature, a systematic review of transtibial prosthetic socket designs by Safari and Meier concluded that TSB sockets exhibited improved weight-bearing, greater suspension and reduced pistoning, which may be, in part, due to the increased peripheral shear from the TSB socket (Safari and Meier 2015). However, extensive experimental data collection is required to validate such a hypothesis.

Differences in the Pareto Front were observed between the virtual people. While the minimum value of fitness function 1 was consistent across the cases at just below 20, the minima of fitness function 2 varied substantially. This result was to be expected based upon the results of the population model where residuum morphology, in particular the residuum profile, had a substantial effect on the pressure over the bony prominences.

A range of genetic algorithms proved effective in performing multi-objective design optimisation of the socket by handling the complex task of simulating the interplay between rectifications on the competing objective functions of the residual limb across the presented design space. In this case the performance of all methodologies was comparable and it could be concluded that the utilisation of several GAs was unnecessary. However, in this case the problem is rather simple to optimise, as 50,000 fitness function calls are sufficient to provide good approximation of the best Pareto Front, and in some cases 20,000 was adequate. The problem has continuous search and objective spaces which further indicates its simplicity (Woldesenbet et al. 2009). However, as the importance of utilising multiple methodologies has been shown by previous researchers (Wang et al. 2018), it is strongly advised here to follow this procedure as good practice until the design space for transtibial prosthetic sockets is better understood. In the future, as more variables and objectives are added to the search space, it is expected that the topology of the design space will change and therefore provide an increasing challenge to resolve the optimal points and require review of the required GA parameters and convergence limits.

The presented multi-objective design optimisation provides an early demonstration of how the speed increases achieved by surrogate techniques enable the socket design process to be framed as an engineering design problem. There are several potential improvements that could be implemented within this process. One such approach may be a dual-level solver where the solver starts with no data, runs a full simulation on a limited population of designs, creates a surrogate from these designs and evaluates the fitness of a substantially larger group across the surrogate. The elite individuals, the fittest individuals in the population which are often defined as the top 10%, would be retained for the next generation and the process repeated. This approach would enable the GA to ignore regions which are clearly sub-optimal, and instead prioritise expensive FE analyses in regions where the minima of the fitness function is more likely to be found. As an alternative approach, to prevent overfitting, the surrogate might be used to generate initial generations, and more expensive FE analyses used at the end to select a preferred design from the options along the Pareto Front.

## Limitations

User satisfaction with the socket is ultimately a subjective measure dependent upon a range of human factors such as comfort, pain thresholds and proprioception arising from a firm, functional prosthesis-skeletal coupling. This means that the predictions of pressure, shear stress and soft tissue strain are not directly related to comfort (Mak et al. 2001). Furthermore, the model would not account for local tissue sensitivities associated with neuromata and soft tissue injuries which could only be identified in limb assessment by the prosthetist. This process might therefore be enhanced by surveying functional and user-reported outcome measures across a population of socket designs.

No direct experimental validation of the underlying relationships between socket design and load transfer predicted by the model in this study has been performed, and such validation evidence must be obtained prior to any clinical evaluation. Pressure and shear sensors (Laszczak et al. 2015) and laboratory-based residuum-socket simulators (McGrath et al. 2017) measure the interaction between the residual limb and socket and could be used to reinforce the findings of this study. As the model uses invented residual limb shapes with thousands of socket designs, it is clearly infeasible to perform experimental validation upon any more than a limited subset of data points in this model. However, in future, a limited number of key socket designs should be tested to validate its conclusions. Furthermore, the population-based surrogate model only characterises a simplified representation of the variability which exists across the population. As discussed previously (Steer et al. 2019), a practical application of these tools requires further data to construct the surrogate model, for example variation in femoral or patella geometry, bone and liner material properties, as well as dynamic load cases. Some confidence is provided by corroboration with literature reports of pressure predictions across the limb between 30 and 100 kPa during gait for TSB sockets (Al-Fakih et al. 2013; Beil et al. 2002; Beil and Street 2004; Dumbleton et al. 2009) and 25–320 kPa for PTB (Dumbleton et al. 2009; Zhang et al. 1998; Dou et al. 2006), which is consistent with the range of predicted pressures for the FF1-biased sockets in this study.

As the study is an initial investigation into the methods and potential it forms the basis for further investigations that provide a more complex design. In increasing this complexity a number of elements will change. First, the Kriging model itself will become more complex providing some challenges in the use of this model which must be investigated. Second, the design space will change and this will provide a different set of optimisation challenges. In both instances the methods used will need to be evaluated carefully. In the case of more complex design spaces, other surrogates might become more appropriate, such as Deep Reinforcement learners. These are subject to a disadvantage of requiring more input data. For the optimisation, it is likely that the space will become more discontinuous (Sobey et al. 2019), similar to other more complex applications, and this will require algorithms with stronger diversity (Wang et al. 2018): NSGA-II and cMLSGA. There is also likely to be a greater separation between specialist, which will have even further reduced performance compared to the general solvers: NSGA-II, cMLSGA and HEIA.

## Clinical applications

Attempting to use simulations to inform clinical decision-making requires extreme caution, especially when applied to devices which depend upon personalised design to ensure comfort and functional efficacy, as comfort and proprioception are difficult to quantify. Crucially, in prosthetic limb design we would argue that these techniques should not be used in isolation, or substituted for human-facing clinical practice. The expert prosthetist must retain control over socket design, and the presented optimisation approach could be used to provide a ‘first-guess’ rectification map. The prosthetist would then modify this candidate design according to their own clinical reasoning which combines palpation, user feedback and re-evaluation. Other technologies such as real-time pressure measurements and predictions from the previously reported PCA-kriging model (Steer et al. 2019) incorporated with their skill and experience could provide a technology-enhanced limb assessment. This approach will help the community to test the key translational research question in this field: can the clinical application of FEA support the prosthetist’s evidence base and enable delivery of comfortable, highly functional prosthetic limbs to users in a more timely and efficient manner?

## Conclusion

This paper provides a first assessment of the use of multi-objective optimisation in the design of prosthetic sockets. The experiential judgement and skill-based process of prosthetic socket design is framed as a multi-objective engineering design problem. This is achieved by developing parametric models of the residual limb informed by statistical shape modelling techniques and the prosthetic socket incorporating both total surface bearing and patella tendon bearing rectifications, which allow the underlying biomechanical relationships between the residual limb and prosthetic socket to be predicted. In line with experimental data to allow detailed biomechanical validation, the developed methods show substantial potential to be used as part of a more informed socket design process, and provide clinicians with support for selecting from the range of candidate design approaches. The resulting designs correspond with the general forms of the two most popular designs: patella tendon bearing and total surface bearing sockets, at the extremes with a series of variations that result in designs that are a compromise between both in the centre. This results in a difference in pressure of up to 31 kPa over the fibula head and 14 kPa over the residuum tip.

## Data Availability

Supporting data are openly available from the University of Southampton repository at 10.5258/SOTON/D0980

## Footnotes

1 Source codes for all methodologies can be found at: https://bitbucket.org/Pag1c18/cmlsga.
